# Effusive-Constrictive Pericarditis Secondary to Primary Pericardial Lymphoma: A Case Report

**DOI:** 10.4021/wjon449w

**Published:** 2012-04-23

**Authors:** Mauricio Anaya-Cisneros, Matthew S. Tong, Alejandro R. Calvo

**Affiliations:** a Graduate Medical Education - Cardiovascular Medicine Fellowship, Kettering Medical Center, USA; b Internal Medicine Residency Program, Kettering Medical Center, USA; c Dayton Cancer Center, Kettering Health Network , USA

**Keywords:** Pericardial lymphoma, Extranodal NHL, Cardiac tamponade, Effusive-constrictive pericarditis, Rituximab, Liver failure

## Abstract

Few cases of primary cardiac lymphoma (PCL) are found in the literature. We report the case of an 85 year-old male who presented with cardiac tamponade and effusive-constrictive pericarditis secondary to primary cardiac lymphoma involving only the pericardium. There have been no prior published cases with these rare scenarios.

## Introduction

Cardiac tamponade due to lymphomatous involvement of the heart is a dramatic and unusual complication. Non-Hodgkin’s lymphoma that predominantly involves the heart rarely is detected antemortem and in many cases constitutes the immediate cause of death. Most cases reported in the literature are of patients with acquired immunodeficiency syndrome. HIV infected individuals develop non-Hodgkin's lymphoma at a frequency 60 to 100 times greater than expected in the general population. In this report, the clinical course of an immunocompetent patient with isolated cardiac lymphoma is reviewed and compared with similar cases described in the literature. The case is of particular interest because of the unusual development of isolated pericardial involvement with cardiac tamponade and effusive-constrictive pericarditis as the sentinel sign of lymphoma.

## Case Report

This is an 85 year-old immunocompetent male with history of paroxysmal atrial fibrillation on warfarin, sick sinus syndrome with dual chamber pacemaker and diastolic heart failure who presented with approximately one-week duration of progressively worsening dyspnea on exertion. This was initially treated as an outpatient with increasing doses of furosemide, but eventually his symptoms worsened to occurring at rest and were associated with orthopnea and paroxysmal nocturnal dyspnea. He denied any chest pain. When he presented to the emergency department he had a respiratory rate of 30 breaths per minute on supplemental oxygen, blood pressure 115/60 mmHg, pulse of 60. There was no pulsus paradoxus, jugular venous distention, friction rubs, or S3/S4 gallops. Heart sounds were decreased.

**Figure 1:**
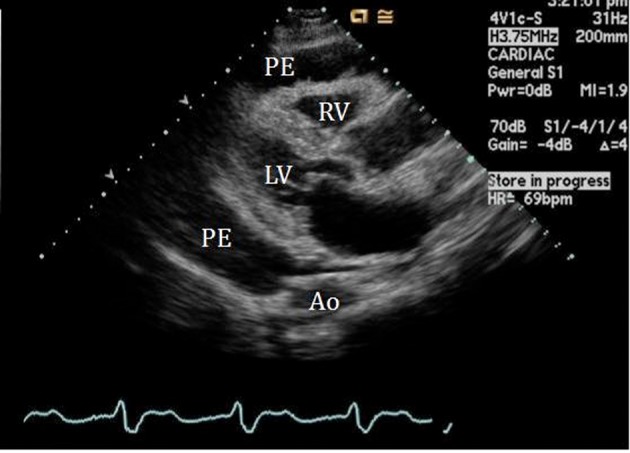
2-D Parasternal long axis view showing large pericardial effusion (PE) surrounding the right (RV) and left ventricle (LV).

**Figure 2:**
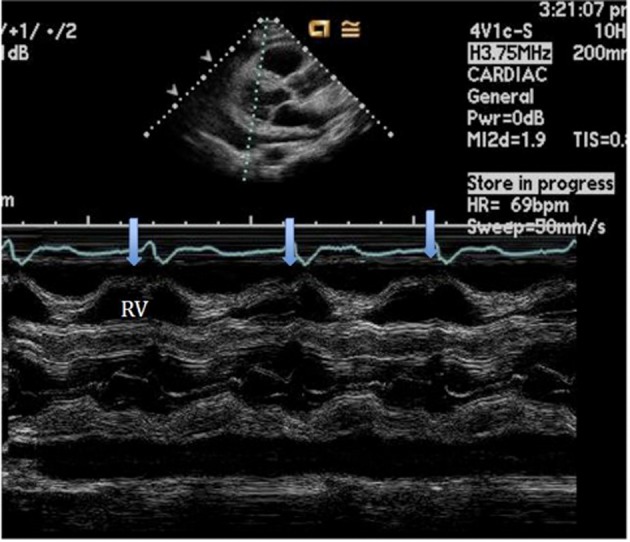
M-mode of the parasternal long axis view establishing the diagnosis of cardiac tamponade showing the collapse of the RV during diastole.

**Figure 3:**
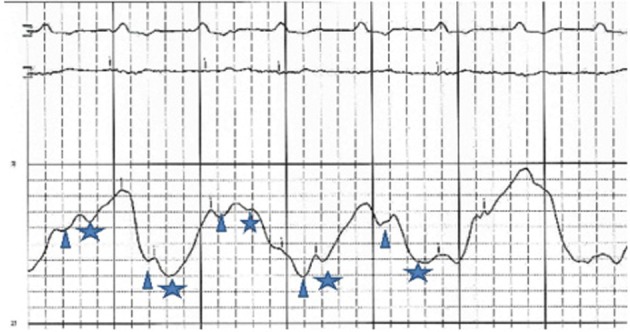
RA pressure before pericardiocentesis: high RA mean pressure at 38 mmHg, X and Y descents are similar. Changes in the baseline reflect patient’s tachypnea.Δ and *represent X and Y descent respectively.

**Figure 4:**
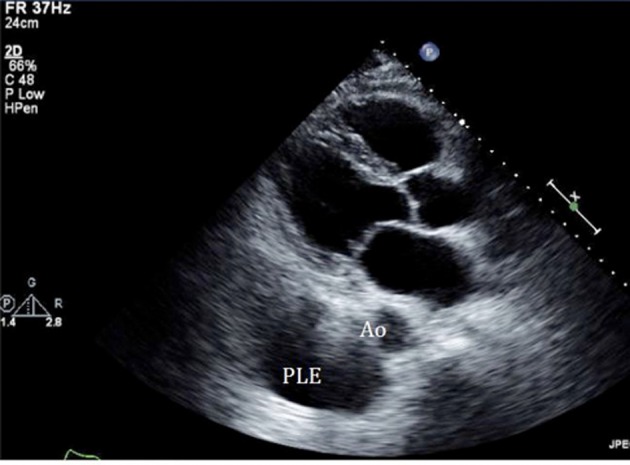
Echocardiogram performed after the pericardiocentesis showed resolution of the effusion, instead pleural effusion (PLE) becomes more evident as an echolucent space below the aorta (Ao).

**Figure 5:**
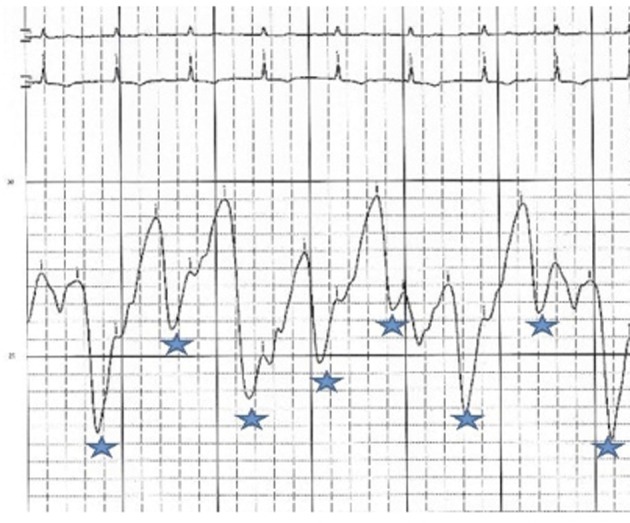
RA pressure after pericardiocentesis: mean RA pressure remains high at 33 mmHg with change in the wave pattern, deep Y descents (*).

An initial complete blood count showed a mild anemia with hemoglobin of 11.2 g/dl. An anterior-posterior chest x-ray showed bilateral interstitial edema with pleural effusions. A transthoracic echocardiogram was performed and found a large pericardial effusion (Fig. 1) with tamponade physiology (Fig. 2). No intracardiac masses were noted. A right heart catheterization was also performed. The initial right atrial (RA) and intrapericardial pressures were elevated at 38 and 31 mmHg, respectively (Fig. 3). The patient underwent urgent pericardiocentesis after warfarin reversal with vitamin K and fresh frozen plasma. Approximately 1060 ml of blood-tinged fluid were removed. After pericardiocentesis there was no residual pericardial fluid assessed by transthoracic echocardiogram (Fig. 4), despite that the RA pressure remained significantly elevated at 33 mmHg. The RA tracing revealed a prominent Y descent (Fig. 5). Due to the patient’s supratherapeutic INR (international normalized ratio) and transient neurologic deterioration from sedation, simultaneous right and left heart catheterizations were not performed. The hemodynamics were compatible with effusive-constrictive pericarditis. Cytology and flow cytometry in the pericardial fluid revealed the presence of monoclonal kappa B-cells, CD 5 negative, CD 10, CD 19, CD 20, and CD 45 positive. Morphologically, the cells were consistent with large B-cell non-Hodgkin's lymphoma. A bone marrow biopsy ruled out systemic lymphomatous involvement. Positron emission tomography (PET) scan confirmed the diagnosis of primary cardiac lymphoma (PCL) showing thickening of the anterior pericardium with an standardized uptake value (SUV) of 5.0. There was no malignant uptake in any other structure such as lymph nodes, other serosal surfaces, or solid organs.

After initial pericardiocentesis, his dyspnea improved only partially, thus a thoracentesis was also performed showing lymphoma cells, likely reflecting a secondary site. His immunophenotype was not consistent with that seen in primary effusion lymphoma in which case there is usually a lack of B-cell -associated antigen expression. HHV-8 testing by immunohistochemistry was negative.

The patient was discharged home in stable condition with partial resolution of his symptoms. A few days later he started outpatient chemotherapy with R-CHOP (rituximab, cyclophosphamide, doxorubicin, vincristine, prednisone). There was no re-accumulation of pericardial fluid on monthly surveillance echocardiograms. After 3 cycles of bio-chemotherapy, a repeat PET scan showed a complete response. Unfortunately the patient became very ill and was not able to continue with chemotherapy. He ultimately died from fulminant liver failure secondary to hepatitis B (HBV) reactivation.

## Discussion

Cardiac tumors are predominantly secondary to metastatic tumors, among them lymphomas. On the other hand, primary cardiac tumors are far less common and only 25% are malignant [1-3]. Primary cardiac lymphomas are extremely rare cardiac tumors, only recently re-emerging due to increasing prevalence of immunocompromised populations, including human immunodeficiency virus (HIV). It is estimated that their incidence is less than 1% of all cardiac tumors [1-3]. The right sided chambers are the most frequently involved sites. In the largest review of cases of PCL from Petrich et al, the order of chamber involvement was RA > right ventricle (RV) > left atrium (LA) > left ventricle (LV); less than 10% of patients were found to have only left sided involvement [1]. Pericardial involvement is estimated to be present in 30% to 58% of these cases, manifested as pericardial effusion or less frequently as cardiac tamponade [1-6]. Solitary pericardial involvement is not only less commonly reported but also challenges the hypothesis by Petrich et al, relating to increased exposure of the right heart to extranodal lymphomas via the thoracic duct emptying into the superior vena cava [1]. Arrhythmias and atrioventricular (AV) blocks are also common (56%); the high prevalence of intra-atrial (41%) involvement might explain the incidence of AV blocks (22%) [1]. Our patient already had a history of paroxysmal atrial fibrillation, sick sinus syndrome, and AV block; at presentation he already had a sequentially atrial and ventricular paced rhythm.

Effusive-constrictive pericarditis is a rare form of pericardial disease that is usually diagnosed after pericardiocentesis. The hallmark is the persistence of an elevated right atrial pressure after drainage of pericardial fluid, traditionally documented by normalization of intrapericardial pressure. In our case we documented by echocardiogram with minimal residual pericardial fluid. Other classic features include reversed X/Y ratio in the atrial pressure tracing after drainage of the pericardial fluid, dip-plateau morphology in the ventricular tracings and accentuation of the interventricular interdependence. Its diagnosis is important as patients might require pericardiectomy for treatment of right sided heart failure [7-10].

Our case is unique from many perspectives. Our patient was an otherwise active and healthy immunocompetent individual. His non-Hodgkin’s B-cell lymphoma was confined to the pericardium on PET scan, and neither the CT of the chest nor the multiple echocardiograms showed any intracardiac masses. To our knowledge there are no reports of effusive-constrictive pericarditis in this selected group of patients. Our patient experienced fulminant hepatic failure secondary to hepatitis B reactivation caused by rituximab-induced immunosuppression. The Centers for Disease Control and Prevention (CDC) recommend routine testing for persons at heightened risk of chronic HBV infection including, but not limited to, persons born in countries with HBsAg prevalence of 2%, injection drug users, and persons infected with HIV (11). His hepatitis B status was unknown prior to immunosuppression with rituximab, and serologic tests were not done as he did not have any risk factors.
